# Regiocontrol of the Bulk Polymerization of Lysine Ethyl Ester by the Selection of Suitable Immobilized Enzyme Catalysts

**DOI:** 10.1021/acs.biomac.4c00497

**Published:** 2024-07-15

**Authors:** Kousuke Tsuchiya, Kayo Terada, Taichi Kurita, Takumi Watanabe, Alexandros Lamprou, Keiji Numata

**Affiliations:** † Department of Chemistry and Biotechnology, School of Engineering, The University of Tokyo, Tokyo 113-8656, Japan; ‡ Biomacromolecules Research Team, RIKEN Center for Sustainable Resource Science, 2-1 Hirosawa, Wako, Saitama 351-0198, Japan; § Department of Material Chemistry, Graduate School of Engineering, Kyoto University, Kyoto 615-8510, Japan; ∥ Innovation Campus Asia Pacific, BASF, Shanghai 200137, China

## Abstract

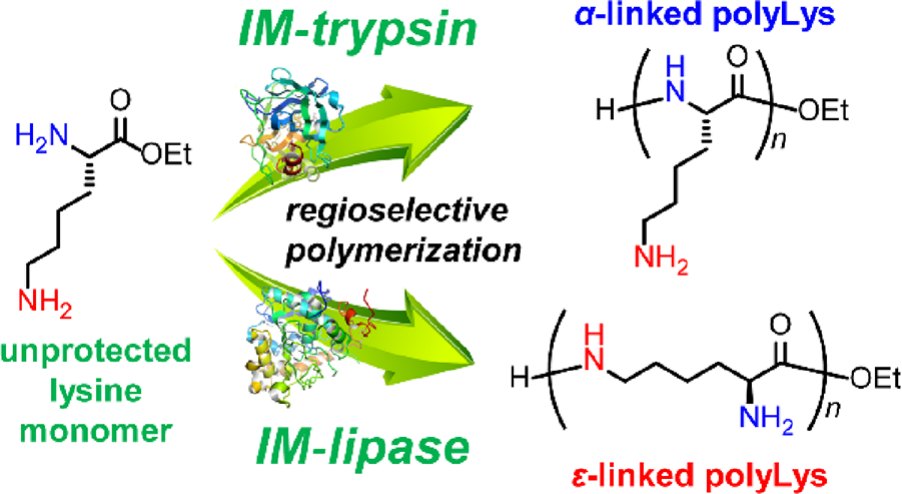

The development of a green and facile method for the controlled synthesis of functional polypeptides is desired for sustainable material applications. In this study, the regioselective synthesis of poly(l-lysine) (polyLys) via enzyme-catalyzed aminolysis was achieved by bulk polymerization of l-lysine ethyl ester (Lys-OEt) using immobilized *Candida antarctica* lipase Novozym 435 (IM-lipase) or trypsin (IM-trypsin). Structural characterization of the obtained polyLys revealed that IM-lipase resulted solely in ε-linked amide bond formation, whereas IM-trypsin predominantly provided α-linked polyLys. Optimization of the conditions for the bulk polymerization using immobilized enzymes resulted in high monomer conversion and a high degree of polymerization, with excellent regioselectivity. Molecular docking simulations revealed different binding conformations of Lys-OEt to the catalytic pockets of lipase and trypsin, which putatively resulted in different amino moieties being used for amide bond formation. The immobilized enzymes were recovered and recycled for bulk polymerization, and the initial activity was maintained in the case of IM-trypsin. The obtained α- and ε-linked polyLys products exhibited different degradability against proteolysis, demonstrating the possibility of versatile applications as sustainable materials. This enzymatic regioregular control enabled the synthesis of well-defined polypeptide-based materials with a diverging structural variety.

## Introduction

Polypeptides exhibiting sustainable features such as excellent biodegradability and low environmental toxicity have inspired many researchers to develop polypeptide-based polymeric materials to replace conventional petroleum-based materials.^1,2^ The structural variation among polypeptides, particularly the diversity of amino acid sequences, offers a wide range of functional properties for various applications. The functionality of polypeptides predominantly relies on higher-order structures, whose assembly depends on the amino acid sequences and the functional groups on the side chain of amino acid residues. The functional groups on the amino acid residues, the reactivity of which typically causes undesired side reactions, need to be protected during peptide synthesis, such as solid-phase peptide synthesis (SPPS) and ring-opening polymerization of amino acid *N*-carboxyanhydrides (NCAs).^3^ The cost and multistep nature of chemical syntheses involving protection/deprotection steps limit the use of these processes for the preparation of polymeric materials and for pilot-scale production. Thus, the demand for sustainable materials based on functional polypeptides poses an important challenge in the field of facile chemoselective synthesis.

Poly(l-lysine) (polyLys) is a functional homopolypeptide with pendant primary amine groups that endow the material with various functionalities. Depending on the amino group involved in amide bond formation, there are two types of polyLys: those with α-linked backbones and those with ε-linked backbones. Naturally occurring polyLys is produced by some bacteria, such as *Streptomyces* sp., and comprises ε-linkages.^4−7^ ε-Linked polyLys (ε-polyLys) exhibits good biocompatibility and antibacterial properties. ε-PolyLys has been used mainly for cosmetics and food additives, but its applications are diverging, such as in regenerative medicines and gene delivery systems.^8−11^ On the other hand, α-linked polyLys (α-polyLys) can be chemically synthesized via the ring-opening polymerization of side group-protected lysine NCA.^12,13^ The main application of α-polyLys is as a biocompatible adhesive coating on various substrates used in biotechnology.^14,15^ Block copolymers containing α-polyLys blocks can also be prepared and applied to antibacterial materials and polymeric carriers for material delivery.^16^ Despite the versatility of polyLys, its tedious synthetic process limits its application: its biosynthesis requires fermentation and purification to remove host cell-derived debris,^17^ whereas its chemical synthesis requires protection/deprotection steps.^12,18,19^ Recently, a facile chemical approach for synthesizing polyLys from unprotected lysine was reported.^20,21^ However, peptide bond formation randomly occurred during thermal polymerization, resulting in hyperbranched polyLys with a mixture of α- and ε-linkages.

The enzyme-catalyzed synthesis of biopolymers has attracted tremendous attention as an environmentally benign process for polymeric material production. We have utilized proteases such as papain and trypsin for the protease-catalyzed polymerization of amino acid ester derivatives to synthesize various polypeptides.^22^ Chemoenzymatic polymerization proceeds in aqueous media under mild conditions and liberates only small alcohol molecules while achieving high atom economy. The substrate specificity of enzymes leads to stereo- and regioselective coupling reactions, enabling chemoselective polymerization of amino acid monomers without any protection of the side groups.^23−25^ We and others have reported that protease-catalyzed polymerization proceeds with both protected and unprotected lysine ester monomers,^26,27^ generating α-polyLys and related α-linked lysine-containing polypeptides.^23,28,29^ Although the polymerization proceeds in an α-linkage-selective way, no enzymatic synthesis of ε-linkage-rich polyLys has been reported to date. In addition, only oligomeric polyLys has been obtained, and no quantitative yield of the isolated polyLys has been reported. If either the α- or ε-amino moiety of lysine can be separately and distinctly utilized for amide bond formation in an enzymatically regulated manner, it is possible to synthesize industrially important α- or ε-polyLys materials from an unprotected lysine monomer by a simple and green method.

Recently, some researchers have reported that diethyl l-aspartate can be polymerized in the presence of immobilized lipase (IM-lipase) without any solvent at an elevated temperature.^30,31^ The regioregularity of the resulting poly(ethyl l-aspartate) was first assigned as predominantly β-linkages^30^ but was recharacterized as predominantly α-linkages (up to 94% α-linkage) by Totsingan et al.^31^ Although this report described only the α-linkage-selective synthesis of poly(l-aspartate), we envisaged that selecting suitable enzymatically controlled reactions of a lysine derivative, even a derivative in which the α- and ε-amino groups exhibit the same reactivity, might lead to multiple types of well-defined polyLys with desired regioselective structures with α- and ε-linked amide bonds. In this study, we developed a novel enzyme-catalyzed bulk polymerization system for the regiocontrolled synthesis of polyLys with a high molecular weight in excellent yield. An appropriate choice of immobilized enzymes, trypsin or lipase, allowed us to regioselectively polymerize a lysine ester monomer in either an α- or ε-linkage-selective manner, respectively.

## Experimental Section

### Materials

Immobilized lipase (IM-lipase), *Candida antarctica* lipase B (CALB) supported on an acrylic resin (Novozym 435), was purchased from Sigma-Aldrich (St. Louis, MO, USA) and used as received (EC number 3.1.1.3). The activity was >5000 U g^–1^ by propyl laurate tests. Immobilized trypsin (IM-trypsin) supported on beaded agarose was purchased from Thermo Fisher Scientific (Waltham, MA USA), and the slurry containing agarose gel was used as received (EC number 3.4.21.4). The loading rate of trypsin on the agarose gel was determined to be approximately 6 wt % from the elementary analysis (C: 44.13%; H: 7.09%; N: 1.42%). The activity was >200 U mg^–1^ by *p*-toluene sulfonyl-l-arginine methyl ester (TAME) tests. l-Lysine ethyl ester (Lys-OEt) dihydrochloride salt was purchased from Watanabe Chemical Industries, Ltd. (Hiroshima, Japan) and used for bulk polymerization after desalting to remove hydrochloride. Dimeric (diLys) and trimeric l-lysine (tryLys) samples as model compounds were synthesized by conventional solution synthesis. For all experiments, water was distilled and ion-exchanged prior to use.

### Desalting of Lys-OEt Hydrochloride Salt

Lys-OEt dihydrochloride salt was desalted via the following procedure. Lys-OEt dihydrochloride (5 g) was dissolved in saturated sodium hydrogen carbonate solution (25 mL) in an ice bath. The pH was adjusted to 10.5 by addition of 5 M sodium hydroxide (NaOH) solution. The resultant solution was poured into a 300 mL extraction funnel. Desalted Lys-OEt was extracted from the aqueous solution to an excess amount of chloroform (200 mL × 5). All the materials were cooled beforehand to suppress the thermal condensation of the desalted Lys-OEt during extraction, while the whole process was conducted in the cold room at 4 °C. The organic layer was dried over NaSO_4_ and then concentrated by a rotary evaporator at 10 °C using a vacuum pump, yielding desalted Lys-OEt as a viscous liquid (2.33 g, 66.1%).

### Bulk Polymerization of Lys-OEt Using Immobilized Enzymes

A typical procedure for the bulk polymerization of Lys-OEt using IM-lipase is as follows. Desalted Lys-OEt (200 μL) was placed into a polypropylene microtube, and 10 mg of IM-lipase was added. The bulk polymerization was performed at 40 °C for 24 h by shaking using a ThermoMixer C (Eppendorf, Hamburg, Germany) at 1600 rpm. Afterward, the mixture was cooled to −30 °C to stop the reaction. An aliquot of the crude sample was taken to characterize monomer conversion by reversed-phase high-performance liquid chromatography (RP-HPLC), regioselectivity by ^1^H NMR spectroscopy, and the degree of polymerization (DP) by matrix-assisted laser desorption/ionization time-of-flight mass (MALDI-TOF MS) spectrometry. The mixture was diluted with water and centrifuged to remove IM-lipase. The recovered IM-lipase was washed with water. All the aqueous solutions were mixed and lyophilized, affording polyLys as a white solid. In the case of IM-trypsin, the bulk polymerization was carried out using almost the same procedure above.

### Determination of Monomer Conversion by RP-HPLC

The monomer conversion for the bulk polymerization of Lys-OEt in the presence of immobilized enzymes was determined by RP-HPLC. RP-HPLC analysis was performed on an HPLC system consisting of an autosampler AS-2055, a gradient pump PU-2089, a column oven CO-4060, a UV/vis detector UV-4075, and a quaternary gradient pump PU-2089 Plus (JASCO, Tokyo, Japan). The mobile phase was composed of acetonitrile (eluent A) and Milli-Q water containing 0.1 v/v% heptafluorobutyric acid (HFBA) (eluent B). The polyLys sample solution was injected and eluted with a mixed mobile phase with a linear gradient from A/B = 5/95 to A/B = 45/55 over 30 min at a flow rate of 1 mL min^–1^ at 25 °C. Each peak in the chromatograph was assigned, and the sum of the peak area assignable to polyLys was compared with that of the monomeric peak to determine the monomer conversion.

### Structural Characterization of PolyLys by ^1^H NMR Spectroscopy and MALDI-TOF MS

The regioselectivity of polyLys obtained by the bulk polymerization in the presence of immobilized enzymes was determined by ^1^H NMR spectroscopy. The lyophilized polyLys was dissolved in D_2_O and acidified with hydrochloric acid. ^1^H NMR spectra of the samples were recorded on a Bruker DPX400 spectrometer (Bruker, Bremen, Germany) at 400 MHz. The regioselectivity was estimated by comparing the integral ratio for the proton of α-carbon of α- (4.1–4.3 ppm) and ε-linkages (3.7–3.9 ppm). MALDI-TOF mass spectra of polyLys were recorded with an AutoFlex III Plus spectrometer (Bruker) using α-cyano-4-hydroxycinnamic acid (CHCA) as a matrix dissolved in acetonitrile or methanol.

### Infrared (IR) and Circular Dichroism (CD) Spectroscopy

The IR spectra of polyLys synthesized by the enzyme-catalyzed bulk polymerization were recorded by using an IR Prestige-21 Fourier transform infrared spectrophotometer (Shimadzu Corporation, Kyoto, Japan) with a MIRacle A single-reflection attenuated total reflection unit using a Ge prism. The CD spectroscopic analysis was carried out using a Jasco J-1500 CD spectropolarimeter (JASCO, Tokyo, Japan) using a cuvette with a 1 mm path at 25 °C. The sample solution was prepared by dissolving polyLys in deionized water at a concentration of 0.1 mg mL^–1^. Each spectrum represents the average values of three independent scans from 190 to 260 nm with a resolution of 1 nm.

### Molecular Docking Simulation

All docking simulation studies were carried out using docking program AutoDock Vina version 1.1.2.^32^ For the protein molecules, the crystallographic structures of lipase (PDB ID 4k6g) and trypsin (PDB ID 2ptc) solved at 1.5 and 1.9 Å resolutions, respectively, were used.^33,34^ Before docking simulations, all ligands and water molecules were removed from the PDB files. Polar hydrogens were added to ligands and receptors by using the Hydrogen module in AutoDock Tools version 1.5.6. Then, Gasteiger united atom partial charges and atom types were assigned, and PDBQT files were generated for AutoDock Vina docking. The protein molecules were kept rigid, while all of the torsional bonds in Lys-OEt were set free to rotate. A 15 Å docking box around the hydroxy group of Ser105 for lipase or Ser195 for trypsin was defined. Images of the binding conformations were prepared using PyMOL 1.8.5.

### Recycle Tests for the Bulk Polymerization Using Immobilized Enzymes

A typical procedure for recycle tests of the IM-lipase-catalyzed bulk polymerization is as follows. The as-received IM-lipase (30 mg, 15 wt %) and the desalted Lys-OEt (200 μL) were placed into a polypropylene microtube, and the bulk polymerization was performed at 10 °C for 72 h according to the above-mentioned procedure. After the polymerization, the mixture was diluted with water (1 mL) and centrifuged at 6000*g* for 1 min. The supernatant was removed to isolate polyLys, and the isolation process was repeated twice. The recovered IM-lipase was washed with Milli-Q water (1 mL) twice, and the aqueous phase was completely removed. The desalted Lys-OEt (200 μL) was added to the microtube containing the recovered IM-lipase, and the second round of the bulk polymerization was performed at the same condition. This sequence of the operations was repeated for each recycle test up to 3 times. In the case of trypsin, the same procedure as IM-lipase was repeated up to 6 times. The monomer conversion, regioselectivity, and DP_max_ of the obtained polyLys were estimated for each recycle test.

### Proteolytic Degradation of PolyLys Using Trypsin

An aqueous solution of α- or ε-polyLys (7.5 mg) in Milli-Q water (0.5 mL) was kept at 30 °C, and a solution of trypsin (1 mg) in Milli-Q water (0.5 mL) was added. The final solution was stirred using a ThermoMixer C (Eppendorf, Hamburg, Germany) at 30 °C and 1800 rpm for 12 h. As a control, the same reaction without trypsin was also performed. After the reaction, trypsin was removed by ultrafiltration using a Vivaspin 500 (MWCO: 10,000) at 9000 rpm for 10 min, and the filtrate was lyophilized. The resulting solid was analyzed by gel permeation chromatography (GPC) using Nexera series with a system controller SCL-40 (Shimadzu, Kyoto, Japan). The sample (5 mg mL^–1^) was eluted with 0.5 M AcOH and 0.1 M NaNO_3_ aqueous solution at 40 °C and a flow rate of 1.0 mL min^–1^ using a Shodex column OHpak SB-803 HQ (Resonac, Tokyo, Japan). The sample was detected by an RI detector RID-20A and a photodiode array detector SPD-M40 (Shimadzu). The chromatogram was analyzed by LabSolutions software (Shimadzu) using poly(ethylene glycol) standards.

## Results and Discussion

### Regioselective Polymerization Using Immobilized Enzymes

**Figure 1:**
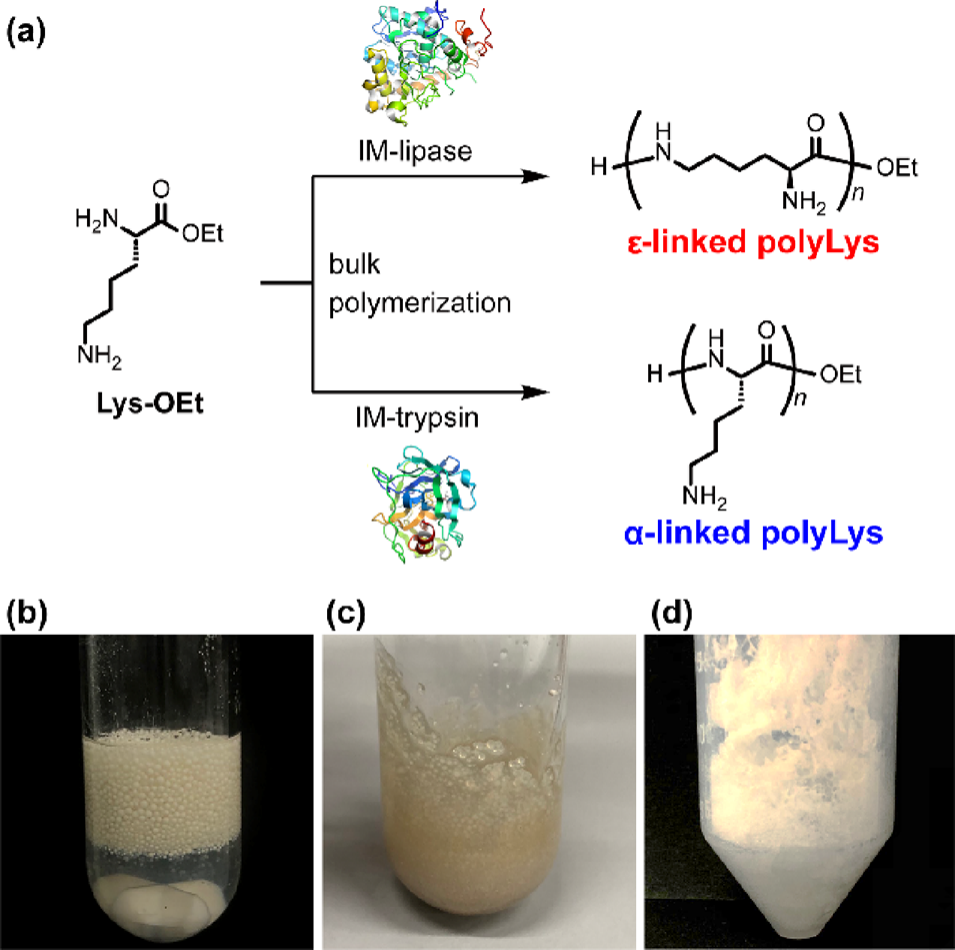
Schematic of the bulk polymerization of lysine ethyl ester (Lys-OEt) in the presence of immobilized enzymes affording α- or ε-linked polyLys (a). Photographs of the reaction mixture before (b) and after (c) bulk polymerization using IM-lipase (15 wt % loading) and photograph of the isolated polyLys (d).

Bulk polymerization using immobilized enzymes has been extensively demonstrated for polyester synthesis, where IM-lipase (most commonly Novozym 435) has been used for the ring-opening polymerization of cyclic ester monomers.^35,36^ Some researchers have also reported that IM-lipase can be used for the synthesis of α-linkage-rich poly(ethyl l-aspartate).^30,31^ These successful examples motivated us to attempt bulk polymerization of a lysine ester monomer using immobilized enzymes (Figure 1a). We used l-lysine ethyl ester (Lys-OEt) as the monomer, which was freshly prepared by desalting a Lys-OEt dihydrochloride salt. The resulting Lys-OEt was a viscous liquid and was easily mixed with the immobilized enzymes (Figure 1b). Two immobilized enzymes were used for this bulk polymerization: IM-lipase (CALB: *Candida antarctica* lipase B, supported on an acrylic resin) and immobilized trypsin (IM-trypsin, supported on an agarose gel), which shows good substrate specificity for basic amino acids. Bulk polymerization was carried out in the presence of the immobilized enzymes (5–60 wt % in terms of the monomer) at ambient temperature. After polymerization for typically 24–72 h, the reaction mixture was solidified (Figure 1c), indicating that Lys-OEt had been converted to polyLys. The immobilized enzyme was easily separated by filtration, and polyLys was obtained as a white solid after lyophilization (Figure 1d). The polymerization was monitored by RP-HPLC, and structural characterization of the obtained polyLys was performed using ^1^H NMR spectroscopy and MALDI-TOF MS spectrometry.

### Characterization of PolyLys

**Figure 2:**
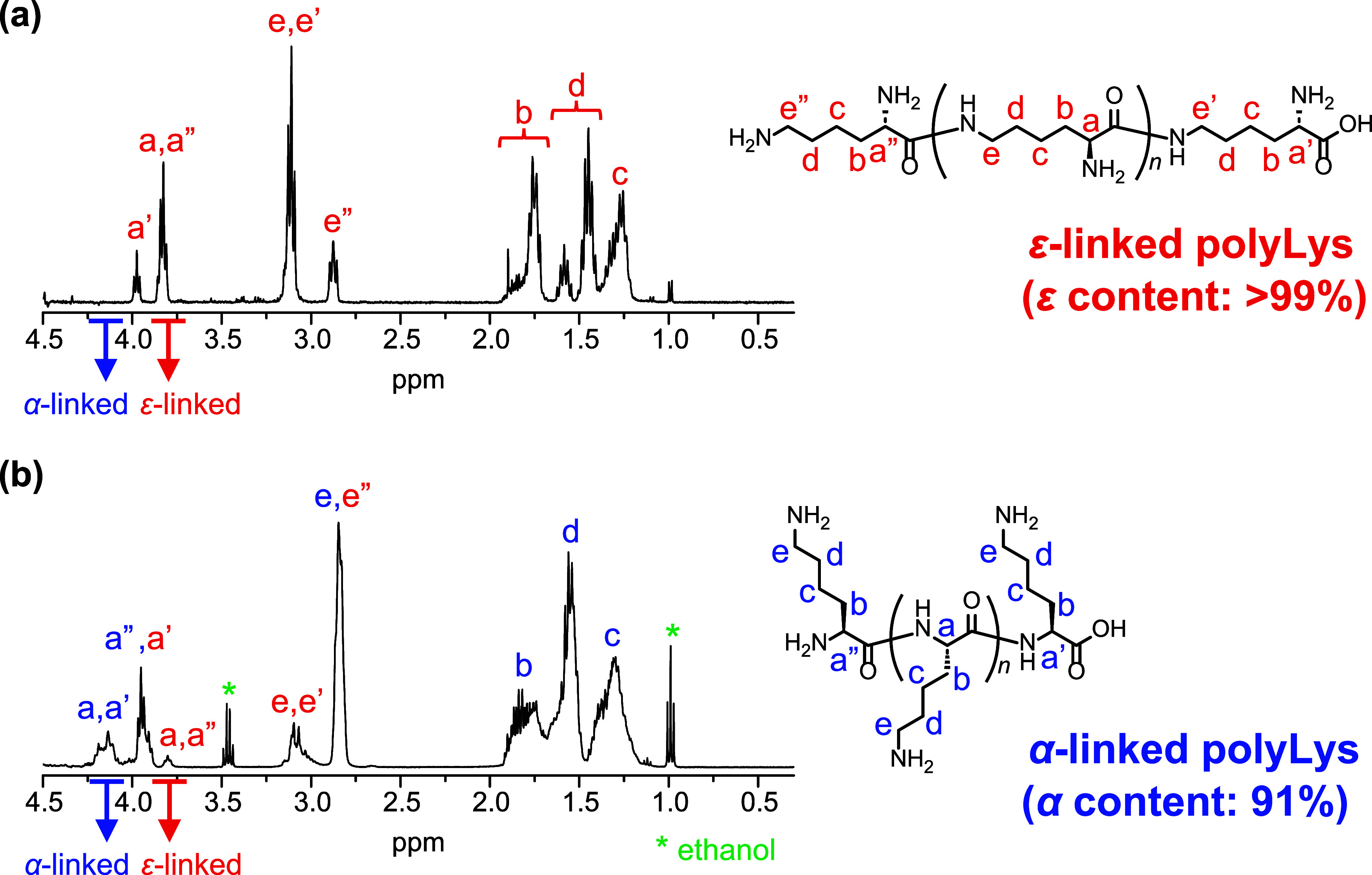
^1^H NMR spectra of polyLys obtained by bulk polymerization using IM-lipase (a) and IM-trypsin (b).

The chemical structures of the obtained polyLys were confirmed by ^1^H NMR spectroscopy. Figure 2 shows ^1^H NMR spectra of the polyLys obtained by bulk polymerization using IM-lipase and IM-trypsin under optimized conditions (discussed in the next section). We found that the chemical shifts of the protons of the α- and ε-carbons were completely different in the spectra of the polyLys products obtained using IM-lipase and IM-trypsin. The peak attributed to the α-proton appeared at 3.7–3.9 ppm for polyLys generated by IM-lipase (Figure 2a), whereas that for polyLys generated by IM-trypsin appeared at 4.1–4.3 ppm (Figure 2b). We synthesized α-linked oligo(l-lysine) as a model compound by solution-phase synthesis to determine the chemical structure of polyLys. In the spectra of the model compounds (Lys dimer and trimer, Figure S1), the peak at 4.1–4.3 ppm was assigned to the α-protons of the α-linked structure. Therefore, the IM-lipase-catalyzed polymerization afforded ε-polyLys with almost quantitative ε-linkage formation (ε content, >99%). In contrast, an α-linkage-rich structure was obtained via IM-trypsin-catalyzed polymerization, and the α-content reached 91% under the optimized polymerization conditions. Notably, the polyLys obtained by thermal polymerization without any enzymes exhibited a random structure with an α/ε ratio ranging from 40/60 to 60/40. Thus, the structural characterization revealed that the immobilized enzymes distinctly regulated the selectivity of the amino group of Lys-OEt during amide formation, affording highly regiocontrolled α- or ε-polyLys depending on the enzyme used.

**Figure 3:**
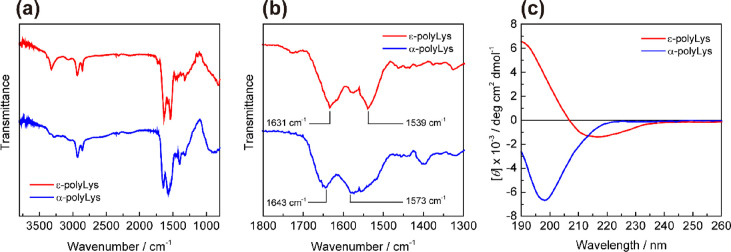
IR spectra of the ε- and α-polyLys obtained by bulk polymerization using IM-lipase and IM-trypsin (a) and expanded spectra for the amide I and II regions (b). CD spectra of the ε- and α-polyLys obtained by bulk polymerization using IM-lipase and IM-trypsin in water (c). The concentration of polyLys was 0.1 mg mL^–1^.

The conformation of the obtained polyLys was also analyzed by IR and CD spectroscopy. The two types of polyLys prepared using IM-lipase and IM-trypsin had almost identical IR spectra, with slight peak shifts in the amide I and II regions (Figure 3a,b). A peak was observed in the amide I region at 1631 cm^–1^ for the ε-polyLys obtained using IM-lipase, corresponding to the carbonyl stretching vibration of amide bonds and reflecting the secondary structures in the solid state.^37^ As determined from the absorption maximum, the amide bonds of ε-polyLys adopted hydrogen-bonded structures similar to β-sheet structures.^37−39^ On the other hand, the spectrum of α-polyLys obtained using IM-trypsin showed an amide I peak with an absorption maximum at 1643 cm^–1^, indicative of more random-coil and/or extended-chain conformations.^37,39−42^ The secondary structures of polyLys in aqueous solutions were investigated by CD spectroscopy. The CD spectrum of ε-polyLys showed an absorption minimum at 215 nm and a maximum at 190 nm, whereas that of α-polyLys showed a minimum at 198 nm (Figure 3c). The CD profile of α-polyLys was indicative of a random conformational propensity in water at neutral pH, as some researchers have reported in the literature.^43,44^ α-PolyLys was reported to exhibit an α-helix structure when the pH was changed from neutral to basic (∼12).^44^ However, the CD profile of the α-polyLys synthesized in this study showed a slight change at pH 12, indicating a propensity for a random conformation (Figure S2). This was attributed to the presence of some amount of short oligomers, as the formation of an α-helix conformation requires a sufficient chain length of α-polyLys.^45,46^ The CD spectra of both ε- and α-polyLys exhibited a Cotton effect, indicating that the stereocenter of l-lysine was maintained during the chemoenzymatic polymerization even when using IM-lipase, which generally does not recognize lysine as a native substrate. Together with the ^1^H NMR results, these findings indicate that chemoenzymatic bulk polymerization afforded two distinct types of polyLys with good control of both the regioselectivity and stereoselectivity.

### Optimization of the Bulk Polymerization Conditions

**Figure 4:**
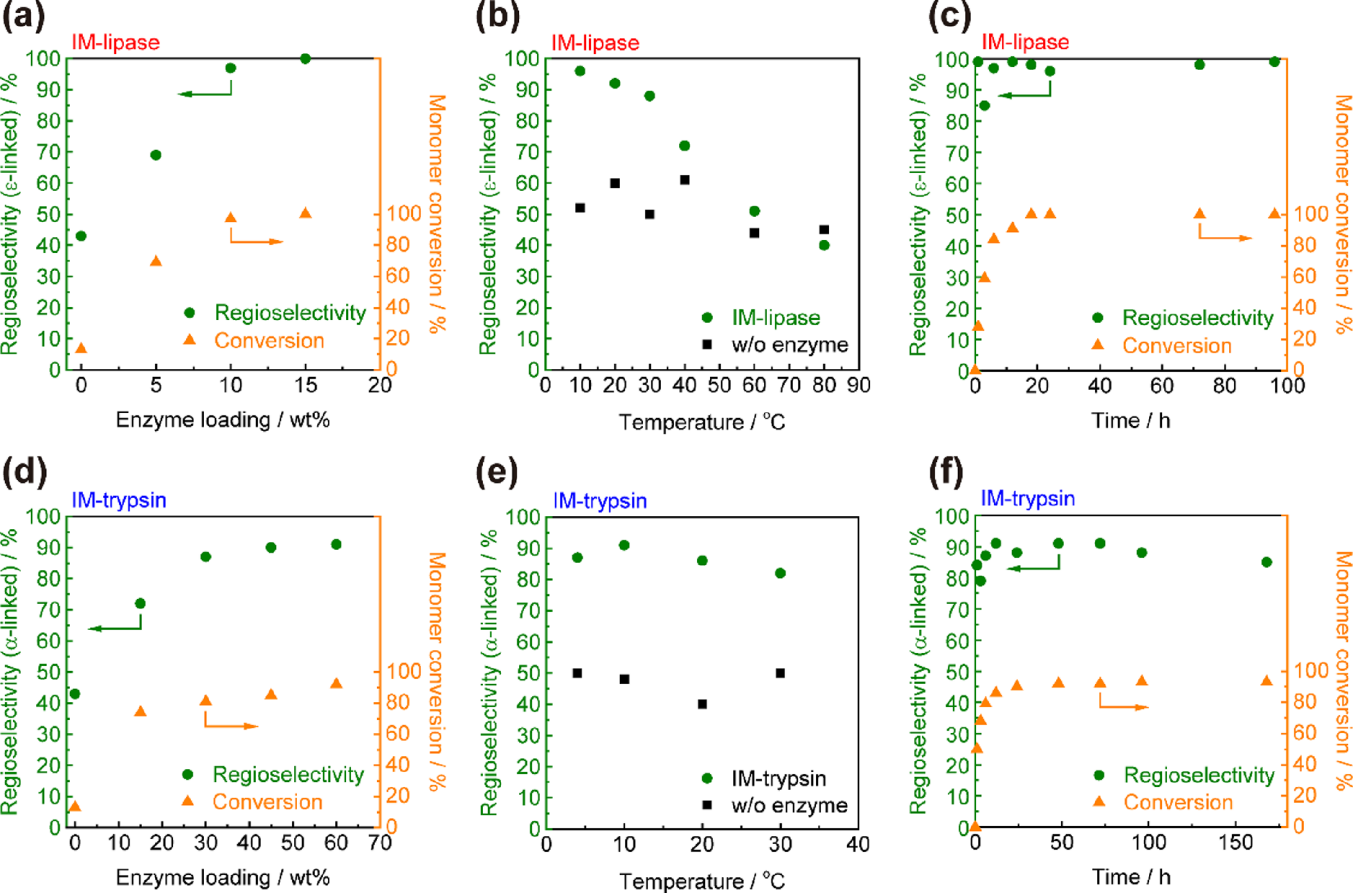
Effect of loading IM-lipase (a) or IM-trypsin (d) on the monomer conversion and regioselectivity of polyLys for bulk polymerization at 10 °C for 72 h. The regioselectivity of polyLys for bulk polymerization using 15 wt % IM-lipase (b) or 60 wt % IM-trypsin (e) at various temperatures for 24 h. Time course study of the monomer conversion and regioselectivity of polyLys for bulk polymerization using 15 wt % IM-lipase (c) or 60 wt % IM-trypsin (f) at 10 °C.

To maximize not only the regioselectivity but also the molecular weight and monomer conversion of polyLys, we investigated the effect of various reaction parameters, including the enzyme loading, reaction temperature, and time, on the bulk polymerization. The monomer conversion was estimated by RP-HPLC analysis (Figure S3). In the case of IM-lipase, we varied the enzyme loading between 5 and 15 wt % for the bulk polymerization of Lys-OEt at 10 °C for 72 h (Figure 4a). Nonenzymatic polymerization at 10 °C afforded polyLys with poor regioselectivity (α/ε content: 57/43) in a low yield of 13%. The addition of 5 wt % IM-lipase dramatically improved the monomer conversion, and the ε-linkage content increased to 69%. The regioselectivity and the conversion increased to 100% as the loading further increased to 15 wt %. Loadings higher than 15 wt % caused ineffective mixing due to the bulkiness of IM-lipase, resulting in no improvement in the regioselectivity and monomer conversion compared with those achieved with a 15 wt % loading. Therefore, we maintained a 15 wt % loading of IM-lipase for further optimization.

Next, the effect of the reaction temperature on the regioselectivity was examined (Figure 4b). IM-lipase (CALB) is a thermally stable enzyme that can catalyze both ester hydrolysis and esterification under harsh conditions, and generally, high temperatures are used for CALB-catalyzed polyester synthesis.^35,36^ In the case of polyLys synthesis, however, temperatures above 40 °C resulted in a random structure with poor regioselectivity. This was probably due to the simultaneous thermal polycondensation of Lys-OEt at high temperatures. By contrast, lowering the reaction temperature gradually increased the ε-linkage content, which reached 96% at 10 °C. Thermal polymerization, which leads to a random mixture of α- and ε-linkages, was effectively suppressed at 10 °C, resulting in a high ε-linkage selectivity. The time course of IM-lipase-catalyzed polymerization was analyzed at 10 °C (Figure 4c). The ε-linkage selectivity was slightly increased by prolonging the reaction time, and almost complete ε-linkage formation (>99%) occurred for more than 72 h. The monomer conversion reached 100% after 18 h.

The molecular weight of the polyLys obtained using IM-lipase was analyzed by MALDI-TOF MS. The MALDI-TOF MS spectra of polyLys showed a series of peaks with an interval of Δ128 *m*/*z* corresponding to lysine residues (Figure S4). The degree of polymerization corresponding to the maximum peak in the MALDI-TOF MS spectrum (DP_max_) was 22.

The IM-trypsin-catalyzed polymerization of Lys-OEt was also investigated in detail. The commercially available IM-trypsin used in this study was supported on an agarose gel, which was expected to require different optimum conditions from those required for IM-lipase supported on an acrylic resin. We studied the effect of the loading of IM-trypsin on the regioselectivity of polyLys (Figure 4d). When 15 wt % IM-trypsin was used, the resulting polyLys exhibited an α-linkage propensity but had a poor selectivity of 72%. The α-linkage selectivity gradually increased with an increasing loading of IM-trypsin and reached 90% at a 60 wt % loading. A trend for the reaction temperature similar to that observed for the polymerization using IM-lipase was observed for the polymerization using IM-trypsin (Figure 4e). Specifically, lowering the temperature to 10 °C slightly improved the α-linkage selectivity. In addition, the DP_max_ of polyLys was greatly improved by lowering the temperature to 10 °C (Figure S5a). Thermal polymerization without IM-trypsin was suppressed at lower temperatures, as revealed by DP_max_, indicating that IM-trypsin clearly catalyzed regioselective α-linked amide bond formation at lower temperatures to afford high-molecular-weight polyLys.

**Figure 5:**
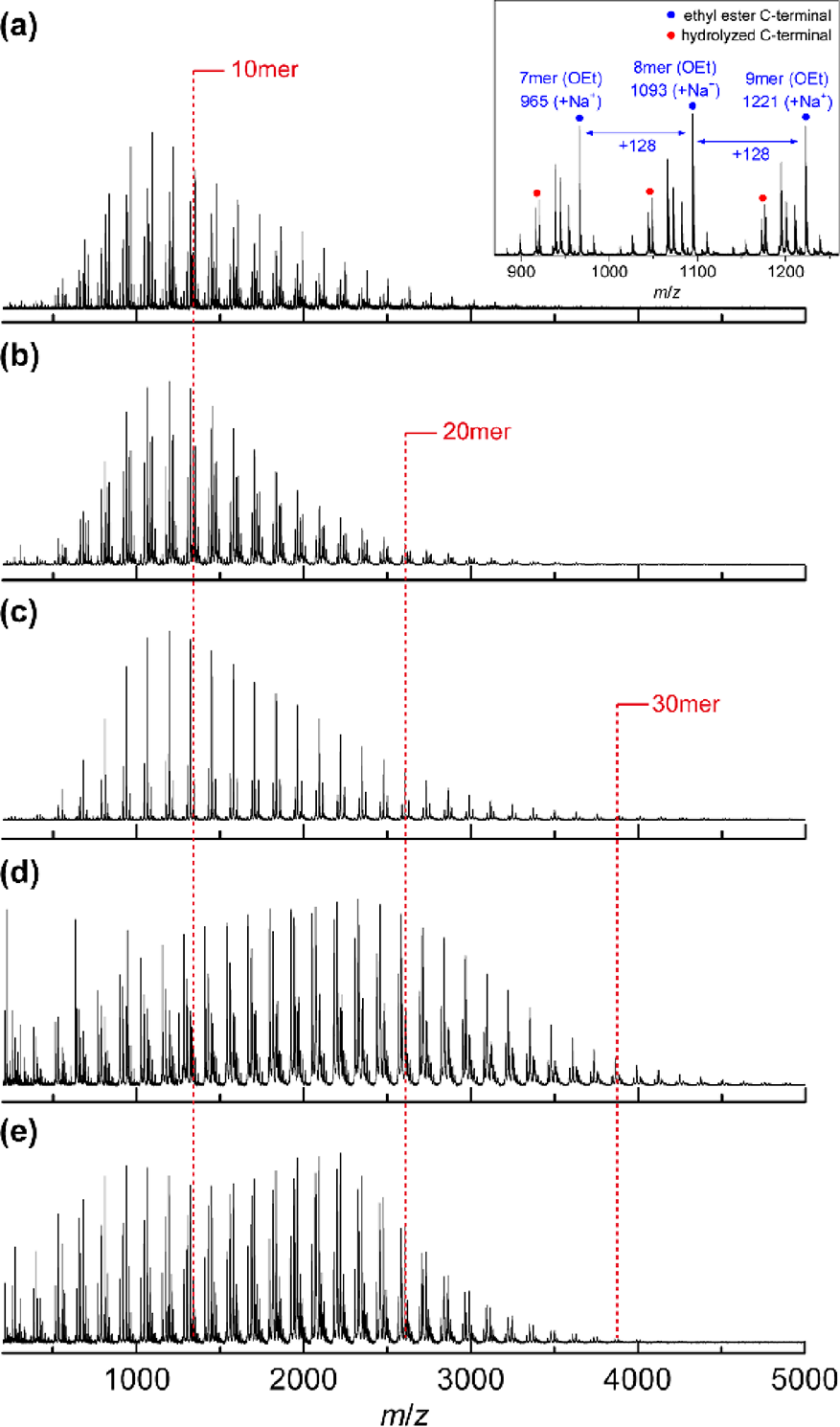
MALDI-TOF MS spectra of the polyLys obtained by bulk polymerization of Lys-OEt in the presence of IM-trypsin (60 wt %). The polymerization was carried out at 10 °C for (a) 24, (b) 48, (c) 72, (d) 96, or (e) 168 h. The inset shows the expanded spectra at 24 h.

The time courses of the regioselectivity and conversion were analyzed for the bulk polymerization at 10 °C using 60 wt % IM-trypsin (Figure 4f). The regioselectivity of polyLys remained almost constant, with a high α-linkage content of approximately 90%, even for a prolonged reaction time of up to 168 h. A monomer conversion of 89% was achieved after 24 h of reaction and gradually increased with an increasing reaction time. The DP_max_ of polyLys was also monitored by MALDI-TOF MS in a time course study (Figure 5 and Figure S5b). The degree of polymerization (DP) ranged from 4 to 25 after 24 h of polymerization (Figure 5a). The major series of peaks with an interval of Δ128 *m*/*z* were assigned to polyLys with an ethyl ester at the C-terminus (inset in Figure 5a). When the reaction time was prolonged, the DP_max_ gradually shifted to higher molecular weights with almost the same peak top during 48–72 h (Figure 5b,c). The distribution of the mass peaks shifted to higher molecular weights, and the DP_max_ increased to 39 after 96 h (Figure 5d). However, the distribution of the peaks slightly shifted to lower molecular weights after 168 h (Figure 5e), indicating that hydrolytic scission of the main chain occurred at extremely long reaction times. This situation caused the peak to shift to both higher and lower molecular weights, resulting in the bimodal distribution as shown in Figure 5e. We assigned several series of peaks in the expanded MALDI-TOF MS spectrum for each reaction time (Figure S6). After 24 h of polymerization, the major peaks were assigned to polyLys with an ethyl ester at the C-terminus. These peaks disappeared after polymerization for more than 96 h, and other series of peaks assignable to polyLys with a carboxylic acid at the C-terminus became more dominant. This result indicated that hydrolysis competed with amide formation during the late stage of polymerization, leading to cleavage of the terminal ester and partial scission of the polyLys main chain after an excessive polymerization time. Overall, the appropriate reaction time for IM-trypsin-catalyzed bulk polymerization was 72 h to avoid undesired hydrolytic side reactions.

### Differences in Substrate Recognition of the Immobilized Enzymes

**Figure 6:**
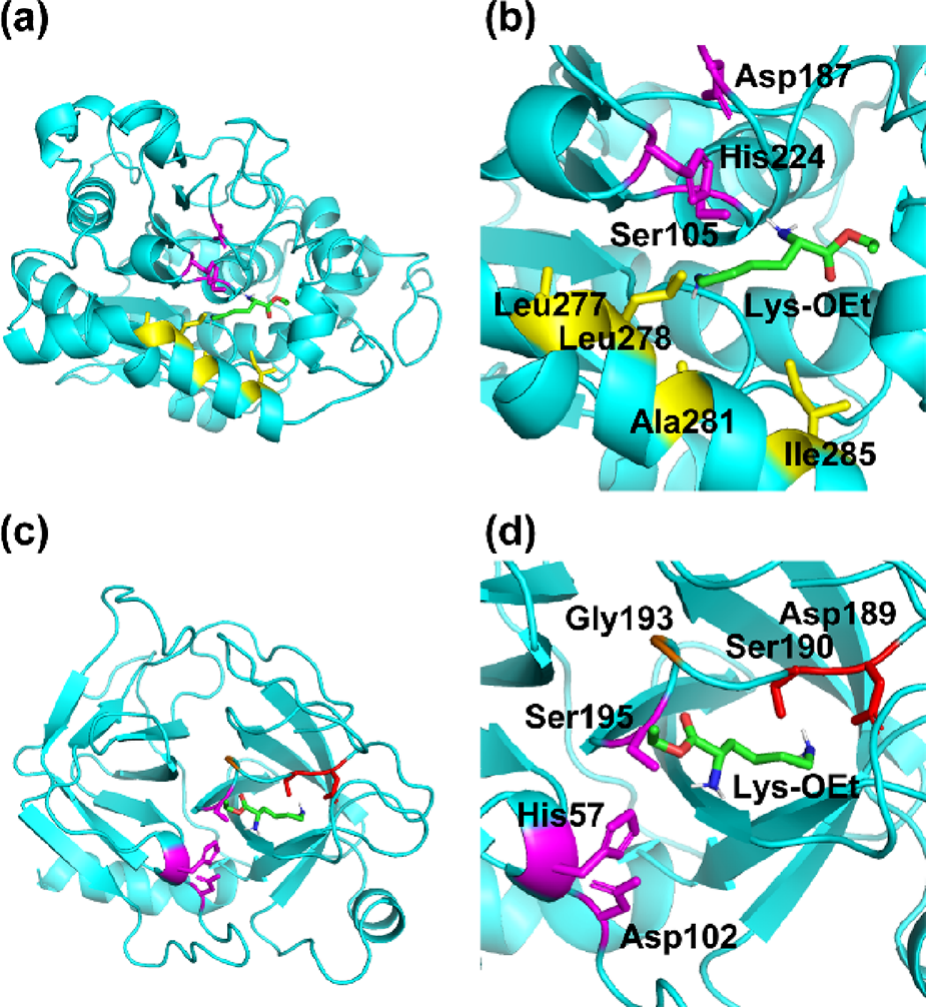
Best binding conformations of Lys-OEt to the catalytic center of lipase (a) and trypsin (c) obtained by molecular docking simulations and magnified images of the catalytic pockets in lipase (b) and trypsin (d). Each enzyme is displayed as a light blue ribbon. The side chains of the catalytic triad in lipase (Ser105, Asp187, and His224) and trypsin (Ser195, Asp102, and His57) are depicted as magenta sticks, and the oxyanion hole of Gly193 in trypsin is shown in orange. The hydrophobic residues (Leu277, Leu278, Ala281, and Ile285) in the catalytic pocket of lipase are shown in yellow, whereas the polar residues (Asp189 and Ser190) in the catalytic pocket of trypsin are depicted in red.

The differences in regioselective amide formation between IM-lipase and IM-trypsin were probably due to the substrate specificity of these enzymes. We carried out molecular docking simulations of the Lys-OEt substrate to these enzymes (lipase or trypsin) to elucidate how Lys-OEt is recognized in the substrate pocket at the catalytic center (Figure 6, Figure S7, and Table S1).

In the most stable conformation, Lys-OEt was located in the catalytic pocket of IM-lipase with the side chain pointed toward the catalytic Ser105 residue (magenta in Figure 6a). The binding free energy of the best binding conformation was −5.0 kcal mol^–1^. The aliphatic alkylamine side chain of Lys-OEt tended to reside close to the catalytic Ser105 residue in the best and third-best binding conformations (Figure 6a,b and Figure S7a,b). The lipase used in this study was CALB, which generally shows distinct substrate specificity for aliphatic long-chain fatty acid derivatives in the catalytic pocket surrounded by hydrophobic residues such as Leu277, Leu278, Ala281, and Ile285.^47−49^ Aliphatic long-chain diamines were also utilized as substrates for CALB-catalyzed polymerization to afford a polyamide by CALB-mediated aminolysis.^50^ In our case, Lys-OEt possessed two primary amine groups at the α- and ε-positions. The substrate pocket in CALB putatively favors the ε-amino moiety, which is more hydrophobic than the α-amino moiety, resulting in highly selective ε-linked amide formation.

Trypsin is a serine protease with substrate specificity for basic amino acid residues such as lysine and arginine.^51^ As expected, the ester group of Lys-OEt was close to the catalytic Ser195 residue, with the carbonyl moiety directed toward the oxyanion hole (Gly193) of the catalytic site in the best binding conformation (Figure 6c,d). The side chain was located near the polar residues, such as Asp189 and Ser190, which confer high affinity to trypsin for basic amino acid residues.^51^ The binding free energy of the best binding conformation was −5.1 kcal mol^–1^. Similarly, the ester group pointed toward the hydroxy group of the Ser195 residue in other structures up to the third-best conformation (Figure 6c,d and Figure S7c,d). This result verified that subsites in the catalytic pocket of trypsin have a high affinity for amino acid residues connected by α-linked amide bonds. Therefore, the IM-trypsin-catalyzed polymerization of Lys-OEt afforded an α-linkage-rich structure.

Overall, the molecular docking simulations indicated that the nature of the binding of Lys-OEt in the catalytic center differed between lipase and trypsin and depended on the environment in the substrate pocket. The side chain of Lys-OEt was assumed to orient along or opposite to the catalytic center in lipase or trypsin, respectively, which resulted in distinct differences in the amino moieties involved in amide bond formation.

### Recyclability of the Immobilized Enzymes

**Figure 7:**
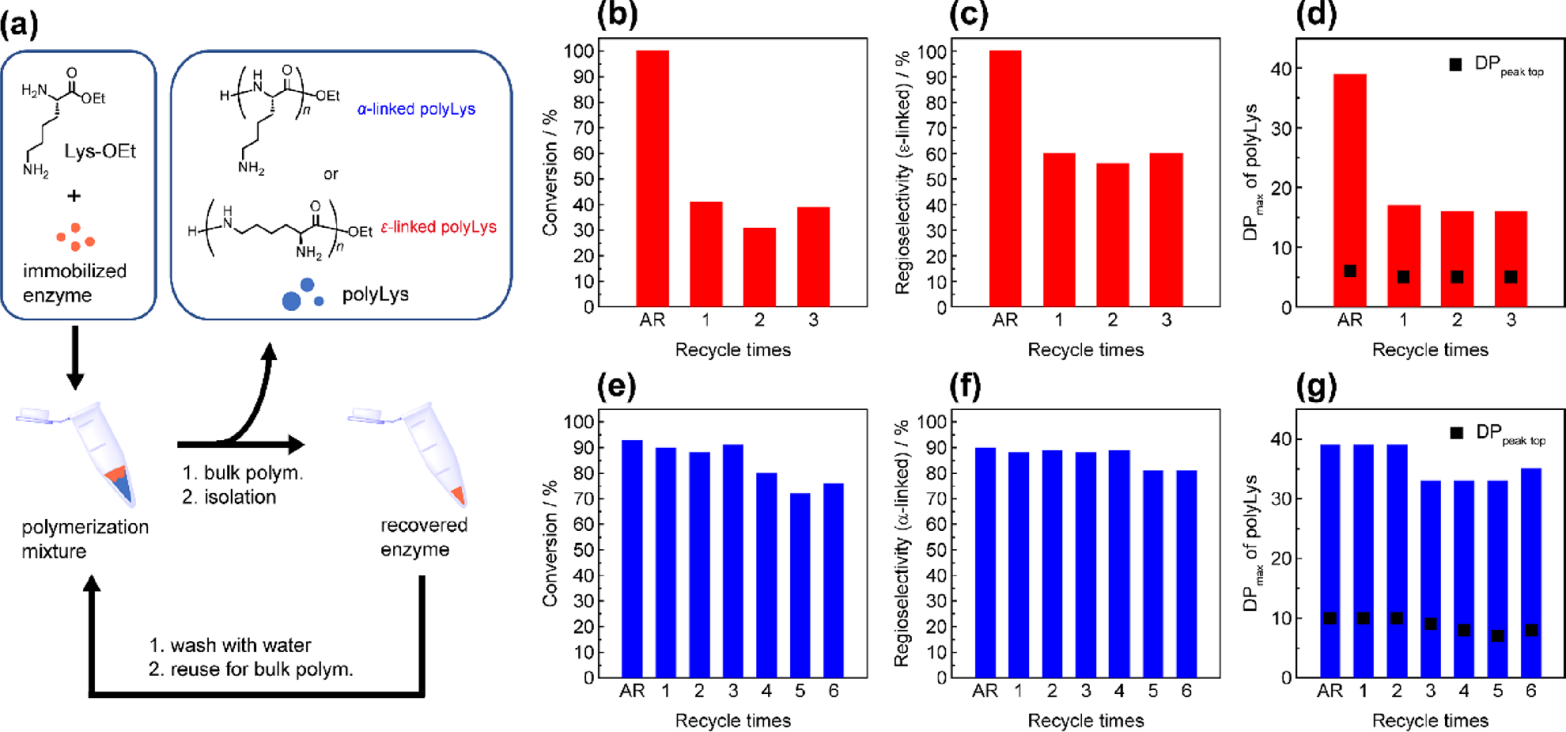
Illustrative image of the enzyme recycling procedure for the bulk polymerization of Lys-OEt (a). Effect of recycled IM-lipase (15 wt %, b–d) or IM-trypsin (60 wt %, e–g) on the bulk polymerization at 10 °C for 24 h: monomer conversion (b,e), regioselectivity (c: ε-linkage, f: α-linkage), and DP_max_ in MALDI-TOF MS spectra (d,g). The degree of polymerization at the peak top in the MALDI-TOF MS spectra (DP_peak top_) was plotted as black squares (d,g). AR: as-received immobilized enzymes.

Using immobilized enzymes for the bulk polymerization of Lys-OEt allowed rapid separation of the enzymes during product isolation. The recovered enzymes were easily washed by rinsing with water and reused for bulk polymerization (Figure 7a). Thus, we performed recyclability tests for both IM-lipase and IM-trypsin for the bulk polymerization of Lys-OEt. After polymerization, the reaction mixture was diluted with water, and the product was isolated by filtration. The filtered immobilized enzyme was washed with water twice and recycled for subsequent polymerization cycles.

The results of the polymerization using recycled immobilized enzymes are summarized in Figure 7. In the case of IM-lipase, the conversion was quantitative, and the ε-linkage selectivity and DP_max_ of the obtained polyLys were 100 and 39, respectively, when the as-received IM-lipase was used under the optimized conditions (Figure 7b–d). However, the use of recovered IM-lipase dramatically decreased both the regioselectivity and DP_max_, with a low conversion of 41%, although bulk polymerization proceeded. We attempted to recover the initial activity by modifying the washing process, such as by rinsing with an aqueous triethylamine solution or methanol, but no remarkable improvement was achieved. In contrast, IM-trypsin showed good reusability for bulk polymerization for up to 6 cycles (Figure 7e–g). The bulk polymerization using as-received IM-trypsin afforded a high monomer conversion of 93% and a high α-linkage selectivity of 90%. During the first and second cycles with recycled IM-trypsin, the conversion, regioselectivity, and DP_max_ were comparable to those achieved using the as-received IM-trypsin. As the number of cycles increased, these values slightly decreased, but the high enzyme activity was still maintained, affording regioselective α-polyLys in high yield. Thus, IM-trypsin could be effectively recycled for multibatch bulk polymerization processes.

The commercially available IM-lipase (Novozym 435) and IM-trypsin used in this study differed in terms of their supporting matrices: a cross-linked acrylic resin was used for IM-lipase, whereas an agarose gel was used for IM-trypsin. The different recyclability behaviors were attributed to differences in the supporting materials. The hydrophobic acrylic polymer used to support IM-lipase is generally applied for esterification in a hydrophobic medium such as organic solvents.^52,53^ A hydrophobic interface is the key for maintaining the activity of IM-lipases. Treatment with water to isolate polyLys might damage the lipase on the hydrophobic support by causing the generation of deactivated forms such as the closed form and dimeric aggregates,^53,54^ thereby decreasing its activity. In contrast, IM-trypsin was supported on a hydrogel based on agarose, which is more tolerable to aqueous treatments, resulting in better recyclability. Therefore, the activity of IM-trypsin was retained after several cycles of bulk polymerization. Further optimization of the supporting matrix and the enzyme recovery process is required to achieve effective recycling of IM-lipase.

### Enzyme Degradability of PolyLys

Both α- and ε-polyLys are biodegradable biopolymers used in different practical applications, but no comprehensive analysis of their biodegradability has been reported to date. We investigated the degradability of the obtained polyLys in the presence of enzymes by performing an in vitro proteolytic degradation assay.

**Figure 8:**
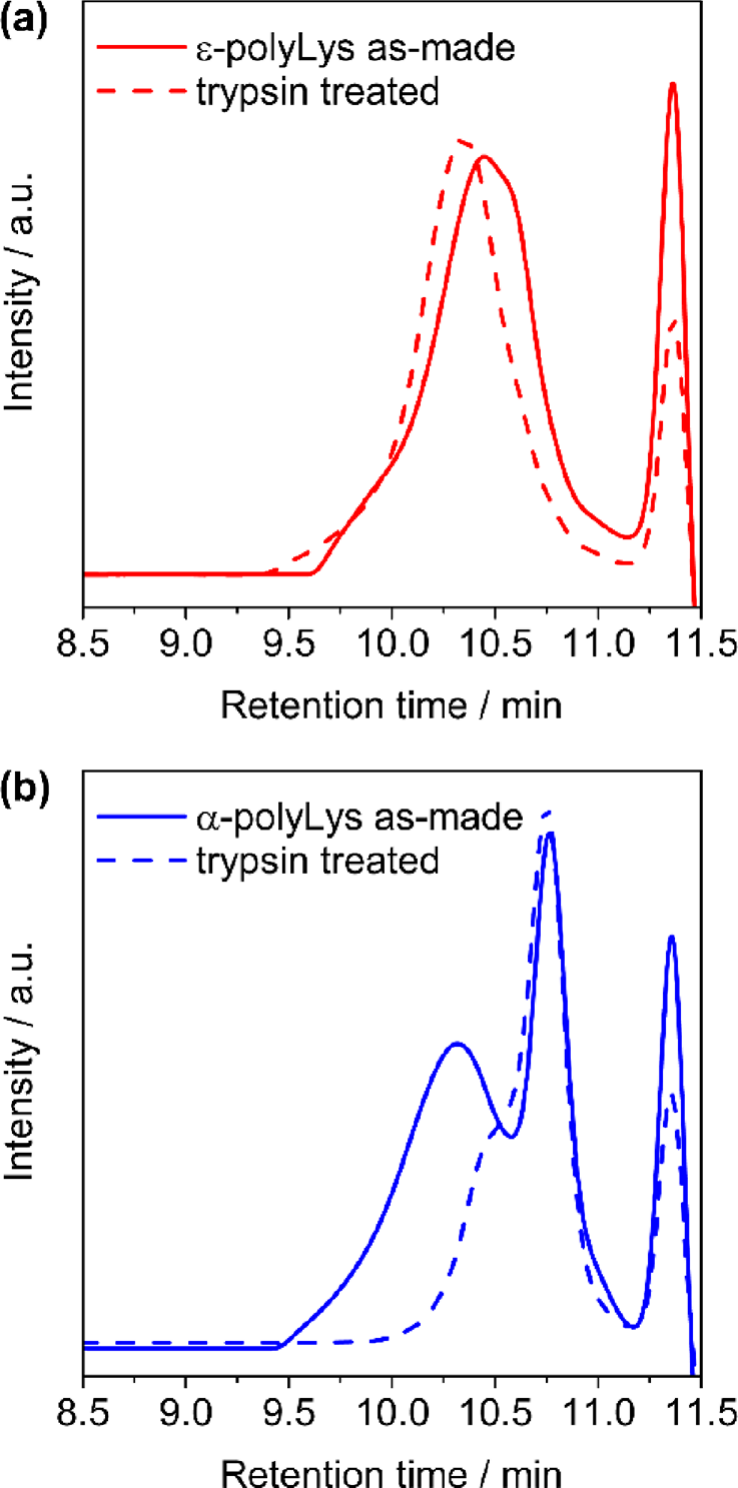
GPC chromatograms of (a) ε-polyLys and (b) α-polyLys before (solid line) and after (dashed line) treatment with trypsin at 30 °C for 12 h. The sharp peak at 11.3 min is a system peak derived from the solvents.

First, we examined the degradability of both α- and ε-polyLys in vitro using trypsin, a serine protease with good affinity for basic amino acids such as lysine. α-Linked amide bonds are expected to be labile to trypsin-mediated cleavage, whereas ε-linked amide bonds are more resistant to proteolytic degradation. The molecular weight distribution of polyLys before and after trypsin treatment was evaluated by GPC, and the results are shown in Figure 8. The GPC chromatogram of ε-polyLys showed a unimodal broad peak at 9.5–11 min (Figure 8a). Notably, the number-average molecular weight (*M*_n_) was estimated to be 530 by using poly(ethylene glycol) standards, but this value was probably underestimated due to the excessive adsorption of cationic polyLys onto the GPC column. The GPC chromatogram of α-polyLys also showed a broad peak at 9.5–11 min (*M*_n_ = 700), as well as a bimodal profile with a sharp peak at 10.8 min derived from residual glycerol contained in IM-trypsin (Figure 8b). After trypsin treatment, the peak for ε-polyLys was almost unchanged. In contrast, the GPC chromatogram of α-polyLys showed a remarkable peak shift to a lower molecular weight. This result clearly indicated that trypsin selectively cleaved the α-linked peptide bonds of polyLys, whereas the ε-linked peptide bond was not recognized in the catalytic pocket of trypsin because nonproteinogenic ε-linked amide bonds resist proteolytic degradation by bacteria, as revealed by in vitro trypsin treatment, except for a few ε-polyLys-producing bacteria that produce specific ε-polyLys-degrading enzymes.^55,56^ Indeed, the moderate biodegradability of ε-polyLys has been reported in living systems,^57^ despite its resistance to proteolysis by trypsin. The difference in proteolytic degradability between α- and ε-polyLys could make these sustainable materials for use in a variety of situations requiring controlled degradation.

## Conclusions

In this study, we developed a bulk polymerization system in the presence of immobilized enzymes to regioselectively generate polyLys from a Lys-OEt monomer. The bulk polymerization proceeded in a facile, easily scalable manner under mild conditions. The immobilized enzymes enabled the synthesis of distinct regiocontrolled structures of both α- and ε-polyLys from unprotected Lys-OEt monomers, eliminating the need for laborious multistep synthetic processes for the precise synthesis of functional polypeptides. Under the optimized conditions, regioselective polyLys with a high DP of up to 39 was obtained in excellent yield. In the case of IM-trypsin, the immobilized enzymes could be reused multiple times, although the ideal recycling process must be optimized for each combination of enzyme and supporting material. In vitro proteolytic degradation revealed that the susceptibility of α- and ε-linked amide bonds to trypsin-mediated degradation is remarkably different. The enzyme-catalyzed synthesis of regiocontrolled polyLys is expected to open avenues for accessing functional (co)polypeptides for various applications.

## Supporting Information Available

^1^H NMR spectra of the model compounds, CD spectra of polyLys, RP-HPLC chromatogram of polyLys, MALDI-TOF MS spectra of polyLys, images of binding conformations obtained by docking simulations, and estimated free energies of the docking simulations (PDF)
