# Supplementary material for: Regiocontrol of the Bulk Polymerization of Lysine Ethyl Ester by the Selection of Suitable Immobilized Enzyme Catalysts
Source: Biomacromolecules. 2024 Jul 15;25(8):5110–20. doi: 10.1021/acs.biomac.4c00497 (PMC11323002; doi:10.1021/acs.biomac.4c00497)
Supplement: Supplementary file 1 — bm4c00497_si_001.pdf [file bm4c00497_si_001.pdf]

Supporting Information

Regiocontrol of the bulk polymerization of lysine ethyl ester by the selection of suitable immobilized enzyme catalysts

Kousuke Tsuchiya,[1,2] Kayo Terada,[3] Taichi Kurita,[3] Takumi Watanabe,[3] Alexandros Lamprou,[4] and Keiji Numata[2,3]**

[1] Department of Chemistry and Biotechnology, School of Engineering, The University of Tokyo, Tokyo 113-8656, Japan.

[2] Biomacromolecules Research Team, RIKEN Center for Sustainable Resource Science, 2-1 Hirosawa, Wako, Saitama 351-0198, Japan.

[3] Department of Material Chemistry, Graduate School of Engineering, Kyoto University, Kyoto Daigaku Katsura, Nishikyo-ku, Kyoto 615-8510, Japan.

[4] Innovation Campus Asia Pacific, BASF, Shanghai 200137, China.

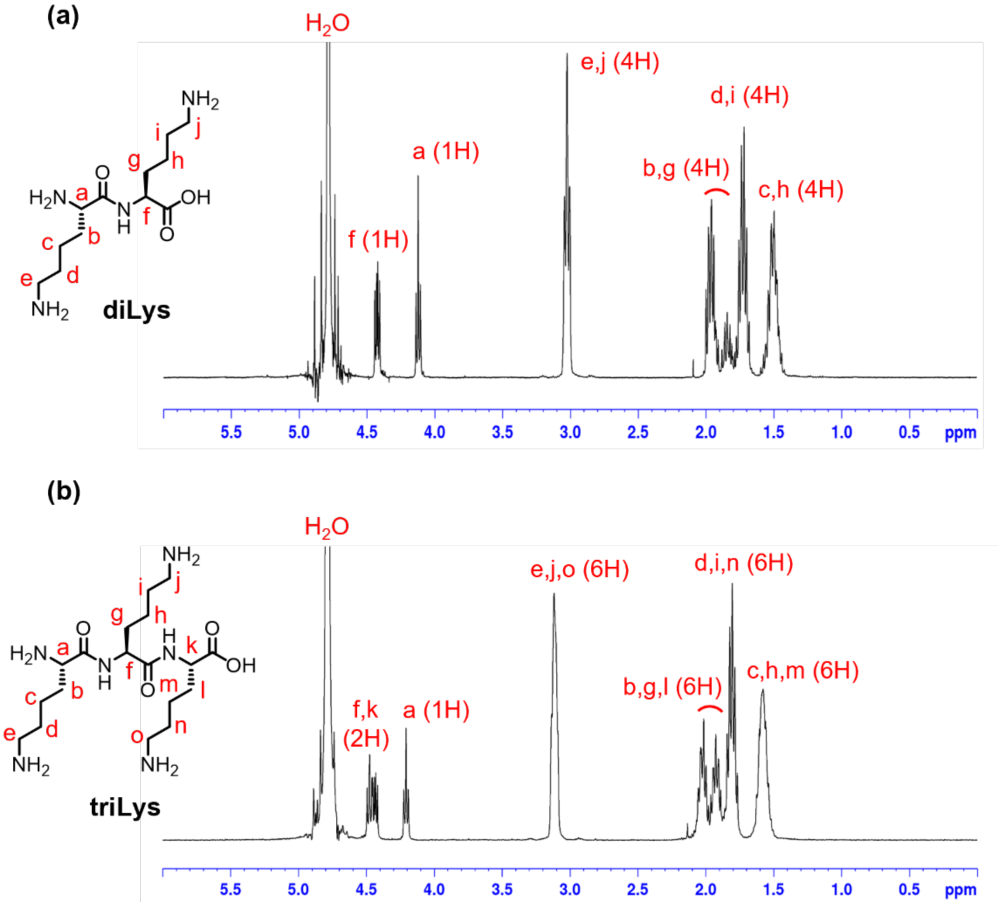

Figure S1. ^{1}H NMR spectra of (a) diLys and (b) tryLys in D_2O.

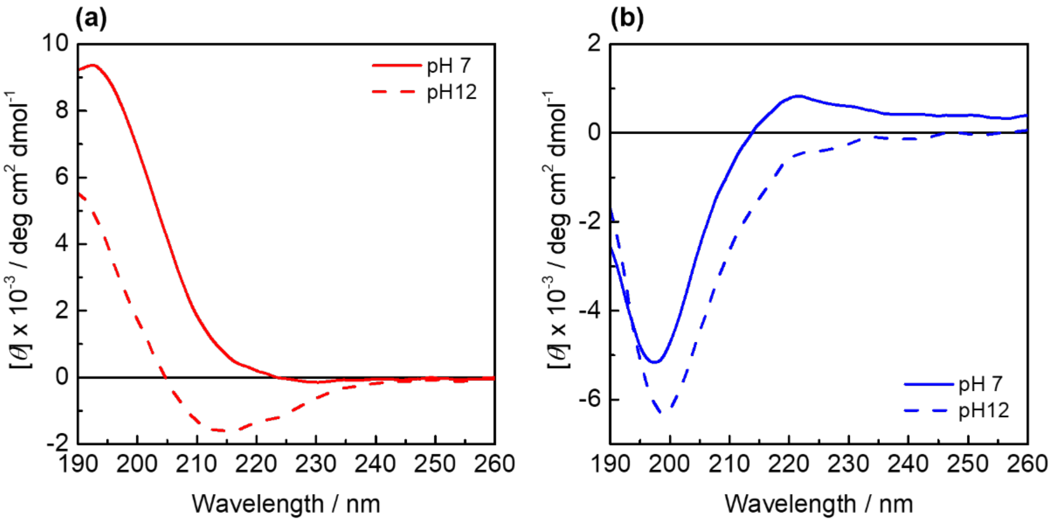

Figure S2. The pH dependence of the secondary structures of (a) α- and (b) ε-polyLys prepared by the bulk polymerization using IM-lipase and IM-trypsin at pH 7 and 12.

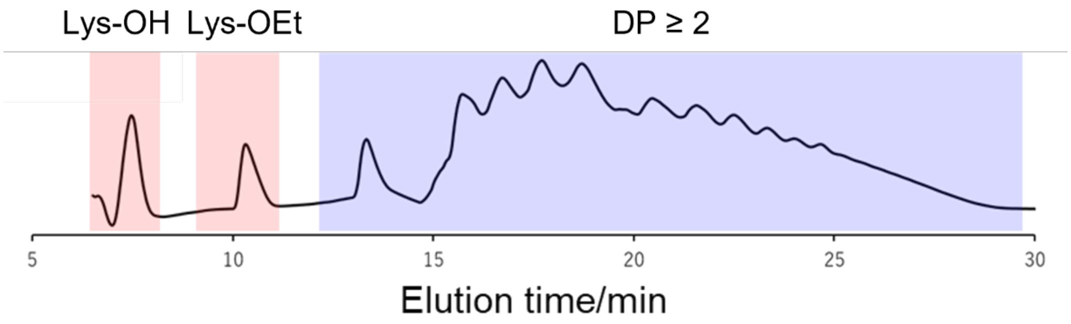

Figure S3. Representative HPLC chromatogram of polyLys obtained by the bulk polymerization using IM-trypsin at 0 °C.

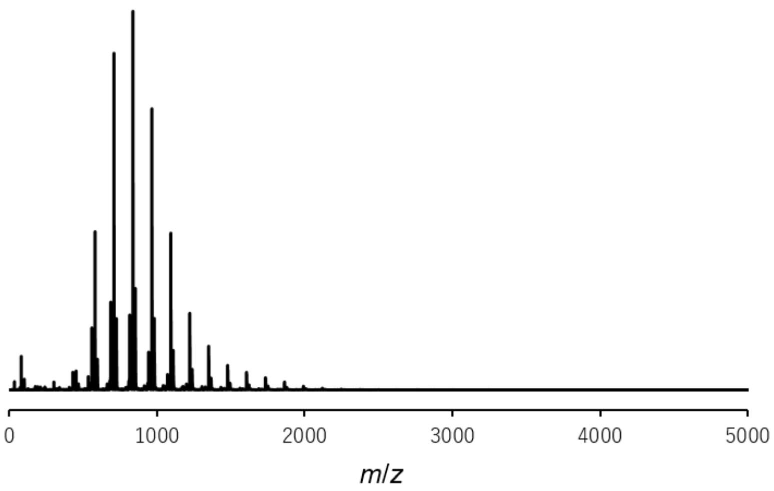

Figure S4. MALDI-TOF MS spectrum of polyLys prepared by the bulk polymerization using IM-lipase (15 wt%) at 10 °C for 72 h.

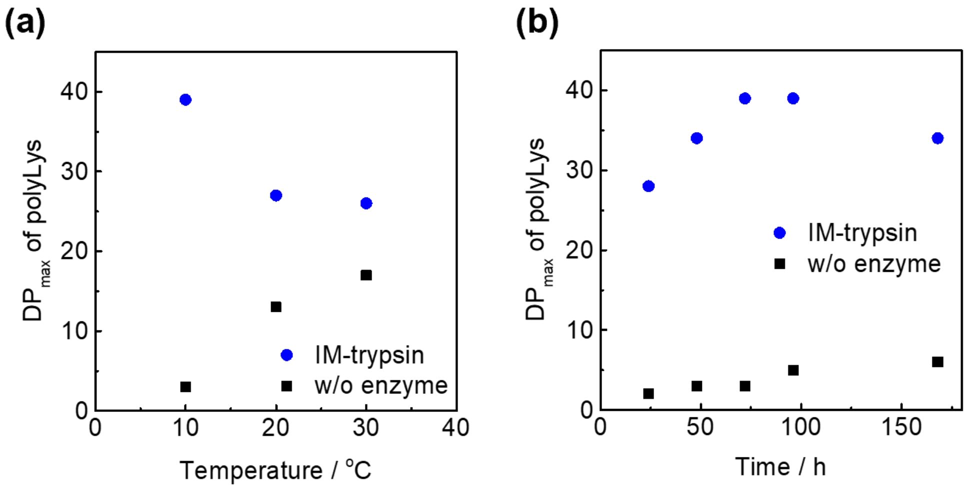

Figure S5. DP_{max} of polyLys determined by MALDI-TOF MS at different polymerization temperatures (a) and different polymerization times at 10 °C (b) for the bulk polymerization using 60 wt% IM-trypsin.

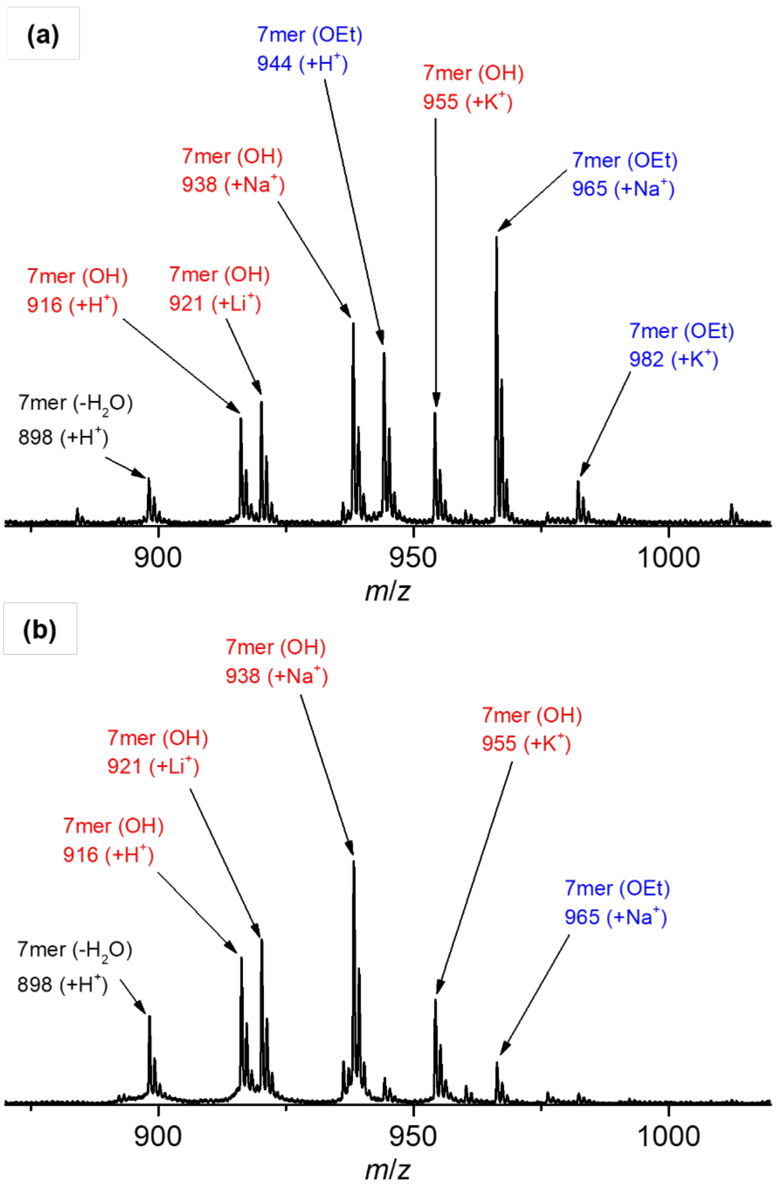

Figure S6. The expanded MALDI-TOF MS spectra of polyLys prepared by the bulk polymerization using IM-trypsin (60 wt%) at 10 °C for (a) 24 h and (b) 168 h. The peaks assignable to PolyLys with an ethyl ester C-terminal are labeled in blue and with a hydrolyzed carboxylic acid C-terminal are labeled in red.

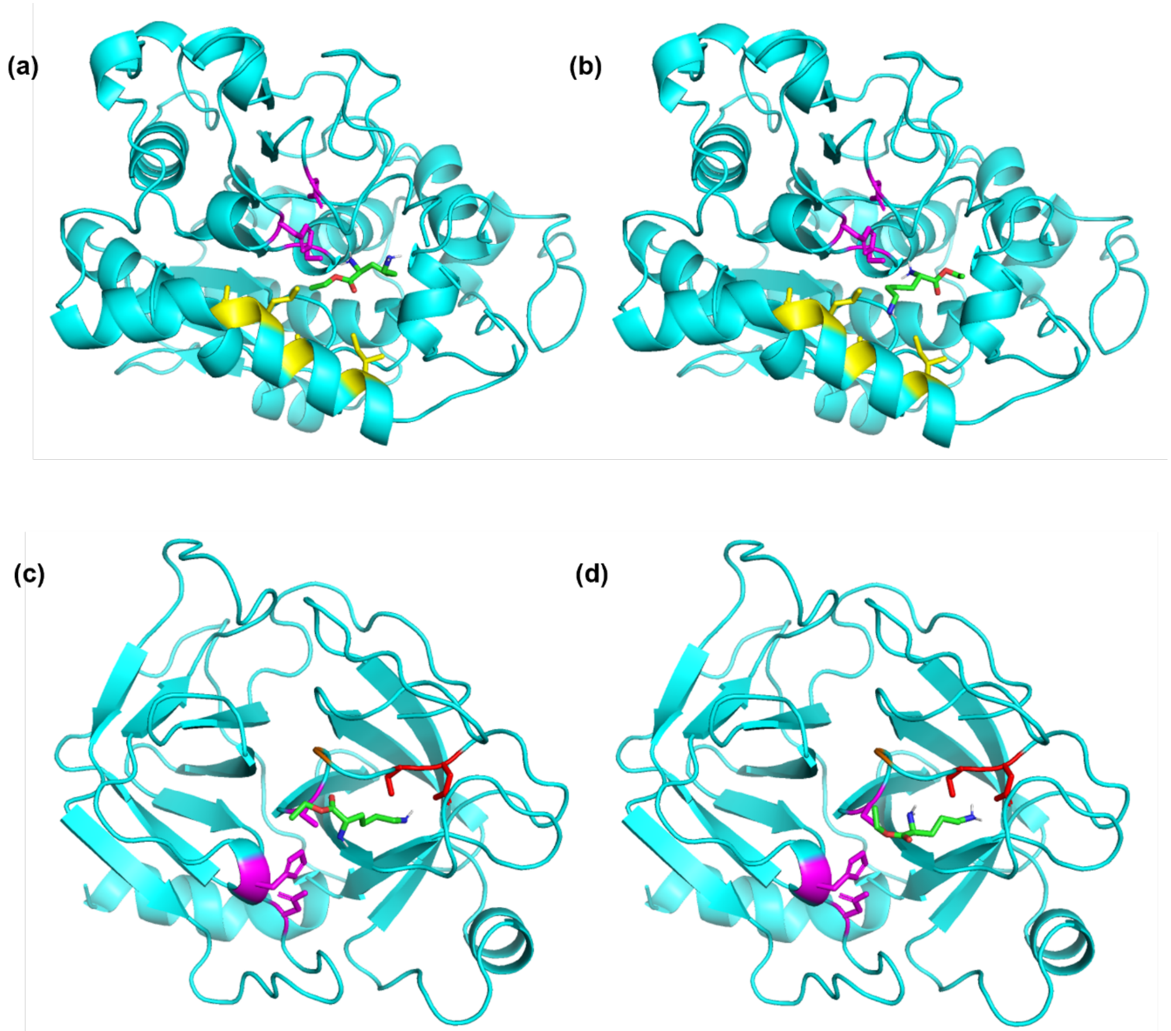

Figure S7. The second (a,c) and third (b,d) best binding conformations of Lys-OEt to lipase (a,b) and trypsin (c,d) obtained by molecular docking simulations. Each enzyme is displayed as a light blue cartoon. The side chains of the catalytic triad in lipase (Ser105, Asp187, His224) and trypsin (Ser195, Asp102, His57) are depicted as magenta sticks and the oxyanion hole of Gly193 in trypsin is shown in orange color. Hydrophobic residues (Leu277, Leu278, Ala281, Ile285) in the catalytic pocket of lipase are highlighted in yellow color (a,b), whereas polar residues (Asp189, Ser190) in the catalytic pocket of trypsin are highlighted in red color (c,d).

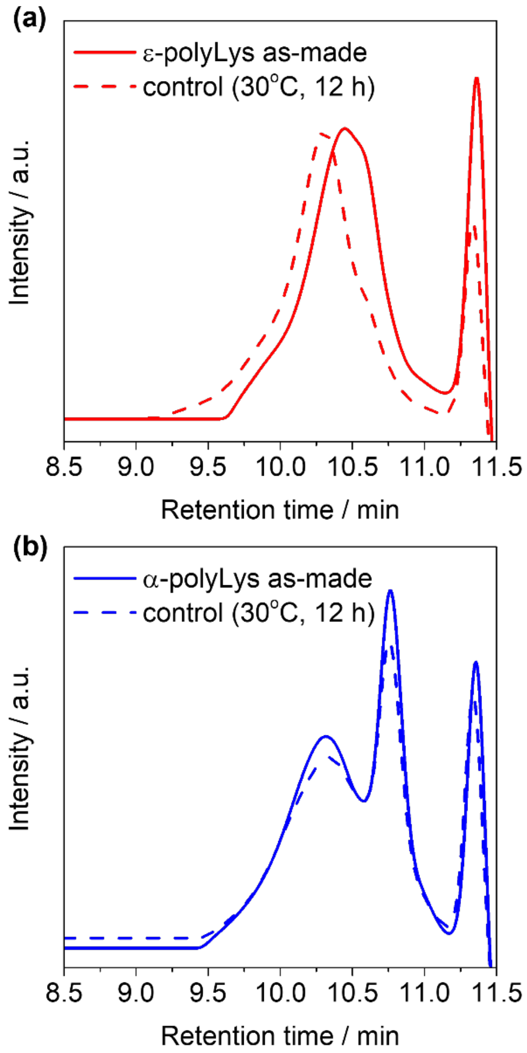

Figure S8. Control experiments for the proteolytic degradation assay using trypsin. GPC chromatograms of (a) ε-polyLys and (b) α-polyLys before (solid line) and after (dashed line) the treatment without trypsin at 30 °C for 12 h. The sharp peak at 11.3 min is a system peak derived from solvents.

Table S1. Estimated binding free energy of Lys-OEt to lipase and trypsin for molecular docking simulations.

	Estimated $\Delta G_{\mathrm{binding}}$ (kcal mol^{-1})	
Mode	Lipase	Trypsin
1	−5.0	−5.1
2	−4.9	−5.0
3	−4.7	−4.6
4	−4.7	−4.4
5	−4.7	−4.4
6	−4.7	−4.4
7	−4.5	−4.3
8	−4.4	−4.3
9	−4.4	−4.2
